# High Blood Pressure and Changes in the Body Mass Index Category Among Japanese Children: A Follow-Up Study Using the Updated American Academy of Pediatrics Guidelines

**DOI:** 10.7759/cureus.26377

**Published:** 2022-06-27

**Authors:** Tatsumi Hayashi, Rumi Sato, Yuhei Ito, Masayuki Ninomiya, Seiji Tanaka, Kazuo Tamura

**Affiliations:** 1 Nutrition, Faculty of Economics, Shimonoseki City University, Shimonoseki, JPN; 2 Nutrition, Bungoono City Council of Social Welfare, Bungoono, JPN; 3 Department of Pediatrics and Child Health, Kurume University School of Medicine, Kurume, JPN; 4 Department of Pediatrics, Ninomiya Clinic, Yanagawa, JPN; 5 Research Promotion Division, Fukuoka University, Fukuoka, JPN; 6 Internal Medicine, Clinical Hematology Oncology Treatment Study Group, Fukuoka, JPN

**Keywords:** body mass index (bmi), follow-up study, common puberty, childhood hypertension, elevated blood pressure

## Abstract

Background

High blood pressure (HBP) has become a public health issue worldwide. The relationship between high BP and changes in the body mass index (BMI) category in Japanese pubertal children has not yet been examined. To resolve this issue, we examined existing data with a focus on the primordial prevention of high BP signs, including elevated BP, among pubertal children aged 12 and 15 years.

Methods

Height, body weight, and BP data were examined from health checkups of 18,247 children conducted between 1993 and 2000 in the Karatsu Study, which was a cohort of pediatric lifestyle-related disease prevention medical health checkups in Japan. BP and BMI were assessed using the updated American Academy of Pediatrics (AAP) guidelines and Endocrine Society’s clinical practice guidelines definitions, respectively.

Results

Follow-up data were obtained from 7,090 subjects (50.5% boys). Stage 2 hypertension (HTN) was detected in 3% and 2.7% of boys and girls aged 12 years, respectively, and in 2.7% and 1% of boys and girls aged 15 years, respectively. Among children aged 15 years, 1.4% were newly classified with stage 2 hypertension, and 15.6% exhibited improvements to a normal BP. A binomial logistic regression analysis of high BP and BMI category changes revealed odds ratios (OR) in the group with a deteriorated BMI category of 1.51 (95% confidence interval (CI), 1.17-1.94), 2.30 (95%CI, 1.66-3.17), and 6.83 (95%CI, 4.14-11.29) for elevated BP, stage 1 hypertension, and stage 2 hypertension, respectively.

Conclusion

High BP in puberty positively correlated with BMI category changes. Considering the presence of the tracking phenomenon in hypertension, BP monitoring is an essential part of the early strategy for the prevention of lifestyle-related diseases in childhood, and improvements in BP control are crucial in early life.

## Introduction

Elevated systolic blood pressure (SBP) and a high body mass index (BMI) are major universal risk factors for cardiovascular diseases (CVDs) [1]. The worldwide prevalence of hypertension (HTN) in adults is high at 31.1%, and thus, its control is an urgent global issue [2]. The prevalence of childhood HTN has reached 3%-4% and is increasing in the general pediatric population, representing a considerable public health challenge worldwide [3,4].

Strong positive correlations have been reported among high BMI in childhood, elevated BP in adolescence, and the prevalence of HTN [5]. HTN and obesity in childhood are risk factors for CVD in young adulthood, emphasizing the importance of treating HTN in overweight and obese children and attempting effective active weight loss [6].

The updated “Clinical Practice Guideline for Screening and Management of High Blood Pressure in Children and Adolescents” (American Academy of Pediatrics (AAP) 2017) [7] has continued to emphasize the importance of BP measurements in children.

In the United States, the BP threshold has been lowered for adults [8] and children [7]. Furthermore, regular annual BP measurements are recommended from the age of three years [7,9]. In Japan, HTN criteria for children were initially established in 2000 and have not been updated [10]. Limited information is available on high BP (HBP) and HTN in childhood and puberty [11,12]. In Japan, BP measurements are not included among health checkup items performed during each school year [13].

Prehypertensive youths (greater than the 90th percentile) are predisposed to CVD and target organ damage (TOD) [14]. Considering the phenomena of tracking, in which children who are in an HBP category tend to fall in the same category when they become adults, the early detection of HBP in children is important for the prevention of CVD/TOD in children and young adults.

The relationship between HBP and changes in the BMI category in Japanese pubertal children has not yet been examined in a large follow-up study. To resolve this issue, we examined existing data with a focus on the primordial prevention of HBP signs, including elevated BP, among pubertal children.

HBP and BMI classifications have not been explicitly established for children, pubertal children, or adolescents in Japan due to inadequate population-specific cutoff values for BP and BMI.

This follow-up study investigated the relationship between HBP and changes in the BMI category in children aged 12 and 15 years by applying definitions by the AAP (2017) [7] and the Endocrine Society’s clinical practice guidelines (2017) [15].

## Materials and methods

Study design and participants

The present study was based on primary health checkup data from the Karatsu Study performed by the Department of Pediatrics, Kurume University School of Medicine, in cooperation with the Karatsu Region General Healthcare Center and the Boards of Education in the Karatsu/Higashi-Matsuura area. The Kurume University School of Medicine Ethics Committee approved the protocol of the Karatsu Study and the use of existing samples and clinical data (approval number 21210), and written informed consent was attained from all participants and their proxy consenters prior to measurements.

The Karatsu Study was performed annually between 1985 and 2000 on all children of the first (six-year-olds), seventh (12-year-olds), and 10th (15-year-olds) grades of all schools in this area [16,17]. The Karatsu Study was one of a cohort of pediatric lifestyle-related disease prevention medical health checkups in Japan. Height, body weight, and BP were measured during the primary health checkup, and HTN and obesity screening, which were assessed noninvasively as risk factors for lifestyle-related diseases, was simultaneously performed with a cardiovascular health examination. BP measurements were repeated up to the secondary or tertiary checkup.

Data were obtained from 18,247 participants (98.52% of the target population) who participated in primary health checkups between 1993 and 2000. The population comprised 10,348 (5,342 boys and 5,006 girls) participants aged 12 years and 7,899 (4,077 boys and 3,822 girls) participants aged 15 years.

In the present study, the values obtained for 12-year-old children who participated in the primary health checkup were regarded as the baseline, and the follow-up ended three years later at the age of 15 years by tracking participants’ data in the primary checkup. The primary checkup was performed in May each year at each school. Height, body weight, and BP data at the ages of 12 and 15 years were obtained from 7,151 subjects (3,603 boys and 3,548 girls).

Measurements

During the primary health checkup, BP was measured twice by a pediatrician while the participants were seated at rest using the automatic sphygmomanometer BP-103N (Nihon Colin, Co., Ltd., Tokyo, Japan), and the value of the second measurement was recorded. In the Karatsu Study, the 95th percentile by age and sex was adopted as the HBP threshold. Baseline values and HBP percentages in the primary health checkup each year during the survey and reference values by the Japanese Society of Hypertension (JSH) (2019) [10] are shown in the Appendices.

Height was measured at 0.1 cm and body weight at 0.1 kg. BMI (kg/m^2^) was calculated by dividing body weight in kg by the square of height in m and rounding the result to one decimal place. Participants with a reduction in height between 12 and 15 years were excluded (n = 19).

Definitions of BP and physique

Using the AAP 2017 definitions, BP was classified into four categories: normal BP, elevated BP, stage 1 HTN, and stage 2 HTN [7]. At baseline (12 years old), these categories were defined using the SBP and diastolic blood pressure (DBP) percentile table specific to age, sex, and height [7]. In 15-year-old participants, BP was categorized as normal for <120/<80 mmHg, elevated for 120/<80 to 129/<80 mmHg, stage 1 HTN for 130/80 to 139/89 mmHg, and stage 2 HTN for ≥140/90 mmHg [7].

Regarding body types, the BMI cutoff values for Japanese children remain unclear. Therefore, the participants were classified as overweight, obese, or extremely obese [15] using age- and sex-specific growth charts [18] developed by the Centers for Disease Control and Prevention (CDC) in 2000 for individuals aged 2-20 years in the United States by referring to the Endocrine Society’s clinical practice guidelines (2017) [15], while the others were classified into a normal BMI group. Participants with BMI less than the fifth percentile (n = 42) were excluded in consideration of possible effects on BP, and four BMI categories were used in analyses.

Other variables

Height, body weight, BMI, SBP, and DBP measurements were converted to z-scores using the LMS method [19] for the standardization of individual participants’ physiques and BP. The z-scores for each measurement (y-value) of height and body weight were calculated using L (skewness), M (median), and S (coefficient of variation) values appropriate for age and sex with the following equation: z = [(y/M)^L-1^] / (L × S) or z = 1n (y/M)/S if L = 0 [20]. Smoothed L, M, and S values for height and body weight [20] were based on data obtained from the national survey in Japan (2000) [21]. SBP and DBP z-scores were calculated using the procedure shown in Appendix B of the 2004 Fourth Report [8]. BMI z-scores were calculated using the L, M, and S values of CDC growth charts [18]. Changes in height, body weight, BMI, SBP, and DBP between 12 and 15 years were represented as Δ to assess changes in BP and body type.

Statistical analysis

Statistical analyses were performed using SPSS Statistics for Windows version 22.0 (IBM Corp., Armonk, NY, USA). All tests were two-tailed, and p < 0.05 was considered to be significant. Data were subjected to the Kolmogorov-Smirnov test to evaluate the normality of their distribution. The measured values for continuous variables were compared using the Mann-Whitney U and Wilcoxon signed-rank tests, and those for categorical variables using the chi-squared test. Continuous variables were presented as median values (50th percentile), and categorical variables as percentages (%) and 95% confidence intervals (95%CI).

The participants were classified according to changes in the BMI category during the three-year follow-up into a maintained group (those who maintained a normal BMI between aged 12 and 15 years), improved group (those in whom the category improved from extremely obese, obese, or overweight aged 12 years to a normal BMI aged 15 years and those in whom the category improved to obese or overweight), unchanged group (those in whom the BMI category remained the same between aged 12 and 15 years), and deteriorated group (those in whom the category was a normal BMI, overweight, or obese aged 12 years but deteriorated aged 15 years). Each respective variable was compared using Kruskal-Wallis and Dunn-Bonferroni’s multiple comparisons tests.

A binomial logistic regression analysis was performed by the forward selection method using elevated BP as the dependent variable (the BP category became elevated BP (1) and a category other than elevated BP (0)) and the BMI category as the independent variable. According to the BMI category, adjustments were performed for sex, the survey year, and baseline SBP (continuous quantity). According to sex, adjustments were performed for the survey year, baseline SBP, and follow-up BMI (continuous quantity of each variable). The odds ratio (OR) of the BMI category for stage 1 and stage 2 HTN were similarly calculated.

Regarding the relationship between HBP and changes in the BMI category aged 15 years, a binomial logistic regression analysis was performed by the forward selection method using normal BP (0) and elevated BP (1) at the age of 15 years as the dependent variable and changes in the BMI category as the independent variable. ORs were adjusted and statistically processed.

## Results

Basic characteristics

Analyzable follow-up data were obtained from 7,090 children (3,580 boys and 3,510 girls). Participant characteristics are shown in Table 1. The median SBP and DBP at 12 years were both significantly higher in girls; however, at 15 years, they were higher in boys (p < 0.001 for both). At baseline, elevated BP, stage 1 HTN, and stage 2 HTN were detected in 8.9%, 16%, and 3% of boys and in 11%, 17.8%, and 2.7% of girls, respectively. At the end of the follow-up, they were detected in 23.4%, 10.1%, and 2.7% of boys and in 14.6%, 4.8%, and 1% of girls, respectively. Obese and extremely obese were more prevalent in boys (p < 0.001).

**Table 1:** Characteristics of Japanese children aged 12 and 15 years in annual health checkups for the prevention of lifestyle-related diseases IQR: interquartile range; CI: confidence interval; BMI: body mass index; SBP: systolic blood pressure; DBP: diastolic blood pressure; HTN: hypertension. †L, M, and S values obtained from anthropometric measurements in the national growth survey of Japan in 2000 were used and substituted into mathematical formulas of the LMS method [20,21]. ‡Measurement value at the end of the follow-up minus that at baseline. §Calculated by referring to the growth charts of the Centers for Disease Control and Prevention and substituting the L, M, and S values into the formula of the LMS method [18]. ¶Calculated and classified according to the Clinical Practice Guideline for Screening and Management of High Blood Pressure in Children and Adolescents (2017) [7]. ††Classified according to the Pediatric Obesity-Assessment, Treatment, and Prevention: Endocrine Society Clinical Practice Guidelines (2017) [15]. ‡‡The Mann-Whitney U test. §§The chi-squared test. *** p < 0.001, * p <0.05, significant difference between baseline and the end of the follow-up, the Wilcoxon signed-rank test.

Characteristics	12 years old (baseline) (n = 7,090)			15 years old (end of the follow-up) (n = 7,090)		
	Boys (n = 3,580) (50.5%)	Girls (n = 3,510) (49.5%)		Boys (n = 3,580) (50.5%)	Girls (n = 3,510) (49.5%)	
	Median (IQR) or % (95%CI)	Median (IQR) or % (95%CI)	p-value	Median (IQR) or % (95%CI)	Median (IQR) or % (95%CI)	p-value
Height (cm)	150.3 (145, 156.1)	151.2 (147.4, 154.9)	0.001^‡‡^	167.6 (163.6, 171.3)^***^	156.1 (152.8, 159.6)^***^	<0.001^‡‡^
Height z-score^†^	0.17 (-0.53, 0.89)	0.20 (-0.41, 0.81)	0.634^‡‡^	0.05 (-0.59, 0.62)^***^	-0.19 (-0.87, 0.53)^***^	<0.001^‡‡^
ΔHeight^‡ ^(cm)				17.8 (13.3, 20.9)	4.3 (2.6, 7)	<0.001^‡‡^
Weight (kg)	41.2 (36.2, 47.4)	43.4 (38.9, 48.8)	<0.001^‡‡^	57 (52, 63)^***^	50.8 (46.7, 56)^***^	<0.001^‡‡^
Weight z-score^†^	0.06 (-0.60, 0.70)	0.23 (-0.34, 0.81)	<0.001^‡‡^	0.11 (-0.44, 0.67)^***^	0.03 (-0.59, 0.68)^***^	<0.001^‡‡^
ΔWeight^‡ ^(kg)				15.8 (12.8, 18.6)	7.5 (4.7, 10.5)	<0.001^‡‡^
BMI (kg/m^2^)	18 (16.8, 19.8)	18.9 (17.4, 20.8)	<0.001^‡‡^	20.2 (18.8, 22)^***^	20.8 (19.2, 22.6)^***^	<0.001^‡‡^
BMI z-score^§^	0.10 (-0.47, 0.71)	0.27 (-0.28, 0.83)	<0.001^‡‡^	0.12 (-0.44, 0.68)^***^	0.28 (-0.24, 0.74)	<0.001^‡‡^
Δ BMI^‡ ^(kg/m^2^)				2.1 (1.3, 3)	1.9 (1.0, 2.8)	<0.001^‡‡^
SBP (mmHg）	107 (100, 117)	109 (102, 118)	<0.001^‡‡^	115 (107, 123)^***^	108 (101, 118)^*^	<0.001^‡‡^
SBP z-score^¶^	0.25 (-0.01, 0.60)	0.33 (0.06, 0.64)	<0.001^‡‡^	0.27 (-0.04, 0.60)	0.14 (-0.11, 0.47)^***^	<0.001^‡‡^
ΔSBP^‡ ^(mmHg）				7 (-1, 15)	0 (-8, 7)	<0.001^‡‡^
DBP (mmHg）	59 (55, 64)	61 (56, 65)	<0.001^‡‡^	63 (59, 68)^***^	62 (58, 67)^***^	<0.001^‡‡^
DBP z-score^¶^	-0.04 (-0.13, 0.05)	-0.03 (-0.13, 0.08)	0.904^‡‡^	0.01 (-0.09, 0.12)^***^	-0.06 (-0.16, 0.06)^***^	<0.001^‡‡^
ΔDBP^‡ ^(mmHg）				4 (0, 9)	2 (-3, 7)	<0.001^‡‡^
BP category^¶^						
Normal BP	72.1 (70.3-73.8)	68.5 (66.6-70.4)	0.001^§§^	63.8 (61.8-65.8)	79.6 (78.1-81.1)	<0.001^§§^
Elevated BP	8.9 (6-12.6)	11 (8.1-14.6)		23.4 (20.6-26.4)	14.6 (11.7-18)	
Stage 1 HTN	16 (13.1-19.3)	17.8 (14.9-21)		10.1 (7.2-13.7)	4.8 (2.1-9.2)	
Stage 2 HTN	3 (0.6-8.3)	2.7 (0.5-8.4)		2.7 (0.5-8.3)	1 (0-10)	
BMI category						
Normal BMI	84.4 (83.1-85.7)	82.9 (81.5-84.3)	<0.001^§§^	85.5 (84.2-86.7)	85.8 (84.5-87)	<0.001^§§^
Overweight^††^	10.1 (7.2-13.7)	13 (10.1-16.4)		9.1 (6.2- 9.1)	10.8 (7.9-14.4)	
Obese^††^	4.6 (1.9-9)	3.6 (1-8.8)		4.3 (1.7-4.3)	2.9 (0.5-8.6)	
Extremely obese^††^	0.9 (0-10.9)	0.5 (0-17.7)		1.1 (0-8.6)	0.5 (0-18.5)	

Changes in the BMI category at baseline and at the end of the follow-up

Variables according to changes in the BMI category during the follow-up are compared in Table 2. The median SBP was normal BP in the maintained and improved groups in boys and girls and was significantly lower in these groups than in the unchanged and deteriorated groups. In boys, the median SBP in the unchanged and deteriorated groups were 126 and 124 mmHg, respectively, and classified as elevated BP. ΔBody weight and ΔBMI were significantly higher in the deteriorated group than in the other groups and were the lowest in the improved group (p < 0.001).

**Table 2:** Comparison of variables in groups classified according to changes in the BMI category at 12 (baseline) and 15 (end of the follow-up) years BMI: body mass index; CI: confidence interval; SBP: systolic blood pressure; DBP: diastolic blood pressure. †Maintained group: those who maintained a normal BMI between aged 12 and 15 years; improved group: those in whom the category improved from extremely obese, obese, or overweight aged 12 years to a normal BMI aged 15 years and those in whom the category improved to obese or overweight; unchanged group: those in whom the BMI category remained the same between aged 12 and 15 years; deteriorated group: those in whom the category was a normal BMI, overweight, or obese aged 12 years but deteriorated aged 15 years. ‡Measurement value at the end of the follow-up minus that at baseline. §The Kruskal-Wallis test. *The highest median values. ABCD: After the Kruskal-Wallis test, a multiple comparison test by Dunn-Bonferroni’s method was performed to identify significant differences (p < 0.05): A: maintained group; B: improved group; C: unchanged group; D: deteriorated group.

Variables	Maintained group^†^	Improved group^†^	Unchanged group^†^	Deteriorated group^†^	p-value^§^
Boys (n = 3,580), % (95%CI)	80.7 (79.2-82.1)	6.2 (3.4-10.3)	7.6 (4.7-11.4)	5.5 (2.7-9.7)	
BMI (kg/m^2^)	19.6 (18.5, 20.9)^BCD^	22.5 (21.3, 23.4)^ACD^	26 (24.5, 29.2)^AB^	25.4 (24.1, 27.7)^AB^	<0.001
SBP (mmHg)	113 (105, 121)^BCD^	118 (109, 125)^ACD^	126 (117, 133)^AB^	124 (116, 132)^AB^	<0.001
DBP (mmHg)	63 (59, 67)^BCD^	64 (60, 69)^ACD^	68 (63, 73)^AB^	68 (62, 72)^AB^	<0.001
ΔHeight^‡^(cm)	18.6 (14.3, 21.3)^*BCD^	16 (11.5, 18.8)^AD^	14.7 (10.5, 18.1)^A^	13 (9.1, 16.9)^AB^	<0.001
ΔWeight^‡^ (kg)	15.6 (13, 18.2)^BCD^	9.6 (5.8, 12.7)^ACD^	19 (15, 22.5)^ABD^	22.8 (19.8, 26.6)^*ABC^	<0.001
ΔBMI^‡^ ((kg/m^2^)	2.1 (1.4, 2.8)^BCD^	-0.5 (-2, 0.7)^ACD^	2.7 (1.8, 3.6)^ABD^	4.9 (4, 6.1)^*ABC^	<0.001
ΔSBP^‡^ (mmHg)	7 (-1, 15)^BD^	2 (-5, 10)^ACD^	7 (-1, 17)^B^	11 (3, 18)^AB^	<0.001
ΔDBP^‡^ (mmHg)	4 (0, 9)^B^	2 (-3, 7)^ACD^	5 (1, 9)^B^	6 (0, 11)^B^	<0.001
Girls (n = 3,510), % (95%CI)	79.8 (78.3-81.3)	7.5 (4.6-11.4)	8.4 (5.5-12.2)	4.3 (1.6-9)	
BMI (kg/m^2^)	20.2 (18.9, 21.5)^BCD^	23.2 (22.2, 23.9)^ACD^	26 (24.9, 27.9)^AB^	25.6 (24.5, 28.2)^AB^	<0.001
SBP (mmHg)	107 (101, 116)^BCD^	111 (104, 120)^ACD^	117 (107, 124)^AB^	116 (105, 124)^AB^	<0.001
DBP (mmHg)	62 (57, 67)^BCD^	63 (60, 68)^A^	65 (60, 69)^A^	66 (60, 70)^A^	<0.001
ΔHeight^‡^(cm)	4.8 (3, 7.7)^*BCD^	2.5 (1.6, 4)^AD^	2.6 (1.6, 4)^AD^	3.6 (2.5, 5.6)^ABC^	<0.001
ΔWeight^‡^ (kg)	7.6 (5, 10.3)^BD^	1.5 (-1.3, 4.1)^ACD^	7.9 (5.8, 10.8)^BD^	14.8 (12, 17.3)^*ABC^	<0.001
ΔBMI^‡^ ((kg/m^2^)	1.9 (1.1, 2.8)^BCD^	-0.2 (1.4, 0.8)^ACD^	2.4 (1.7, 3.2)^ABD^	4.9 (3.8, 5.8)^*ABC^	<0.001
ΔSBP^‡^ (mmHg)	0 (-8, 7)^BD^	-3 (-12, 5)^ACD^	-1 (-9, 9)^BD^	3 (-6, 13)^*ABC^	<0.001
ΔDBP^‡^ (mmHg)	2 (-3, 7)^BD^	1 (-4, 5)^AD^	1 (-3, 5)^D^	4 (-2, 8)^*ABC^	<0.001

Relationship between the BP category and the BMI category at the end of the follow-up

The relationship between HBP and the BMI category at 15 years is shown in Table 3. At 15 years, 1.4% (2.1% of boys and 0.6% of girls) of children were newly classified with stage 2 HTN. The baseline BMI category was above overweight in 49% of these children (data not shown). Furthermore, 4.1% (6.1% of boys and 2.1% of girls) of children were newly classified with stage 1 HTN and 10.7% (14.3% of boys and 7.1% of girls) with elevated BP. Based on the results of the binomial logistic regression analysis, the ORs of stage 2 HTN in obese and extremely obese children were 3.77 (95%CI, 2.24-6.33) and 8.19 (95%CI, 5.04-13.31), respectively. In girls, the ORs of stage 1 and stage 2 HTN were 0.32 (95%CI, 0.24-0.42) and 0.27 (95%CI, 0.16-0.43), respectively, and boys were more likely to newly develop HTN, showing an apparent sex difference (p < 0.001).

**Table 3:** Odds ratio and 95%CI for relationships between high BP and BMI categories and sex at the age of 15 years (end of the follow-up) CI: confidence interval; BP: blood pressure; BMI: body mass index; stage 1 HTN: stage 1 hypertension; stage 2 HTN: stage 2 hypertension; OR: odds ratio. †Classified according to the Clinical Practice Guidelines for Screening and Management of High Blood Pressure in Children and Adolescents (2017) [7]. ‡Classified according to the Pediatric Obesity-Assessment, Treatment, and Prevention: Endocrine Society Clinical Practice Guidelines (2017) [15]. §Adjusted for sex, survey years, and baseline systolic blood pressure (as continuous); however, when sex was used as the independent variable, it was adjusted for survey years, baseline systolic blood pressure, and follow-up BMI (as continuous).

Variables	N	Elevated BP^†^ (10.7%)		Stage 1 HTN^†^ (4.1%)		Stage 2 HTN^†^ (1.4%)	
		OR (95%CI)^§^	p-value	OR (95%CI)^§^	p-value	OR (95%CI)^§^	p-value
		Number of subjects (%)		Number of subjects (%)		Number of subjects (%)	
BMI category							
Normal BMI	6,073	1 (reference)		1 (reference)		1 (reference)	
		631 (10.4)		209 (3.4)		42 (0.7)	
Overweight^‡^	705	1.76 (1.39-2.23)	<0.001	2.46 (1.80-3.36)	<0.001	-	-
		96 (13.6)		54 (7.7)		19 (2.7)	
Obese^‡^	253	1.73 (1.16-2.56)	0.007	2.71 (1.73-4.23)	<0.001	3.77 (2.24-6.33)	<0.001
		32 (12.6)		24 (9.5)		22 (8.7)	
Extremely obese^‡^	59	-	-	2.68 (1.13-6.35)	0.025	8.19 (5.04-13.31)	<0.001
		3 (5.1)		6 (10.2)		15 (25.4)	
Sex^§^							
Boy	3,580	1 (reference)		1 (reference)		1 (reference)	
		514 (14.3)		220 (6.1)		76 (2.1)	
Girl	3,510	0.97 (0.96-0.98)	<0.001	0.32 (0.24-0.42)	<0.001	0.27 (0.16-0.43)	<0.001
		248 (7.1)		73 (2.1)		22 (0.6)	

**Figure 1:**
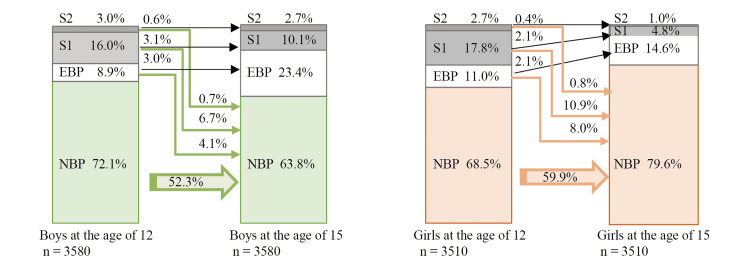
Distribution of blood pressure categories at 12 and 15 years (by sex) NBP: normal blood pressure; EBP: elevated blood pressure; S1: stage 1 hypertension; S2: stage 2 hypertension.

The distribution of BP categories is shown in Figure 1. The BP category improved to normal BP at 15 years in 11.5% (95%CI, 8.4-14.6) of boys and 19.7% (95%CI, 16.7-22.7) of girls. Improvements were observed in 15.6% (95%CI, 13.5-17.7) of all children.

Relationship between the BP category and changes in the BMI category at the end of the follow-up

The relationship between HBP and changes in the BMI category when the follow-up ended at the age of 15 years is shown in Table 4. Based on the results of the binomial logistic regression analysis, the OR of elevated BP was 1.51 (95%CI, 1.17-1.94) and that of stage 1 HTN was 2.30 (95%CI, 1.66-3.17) in the deteriorated group. The ORs of stage 2 HTN were 4.19 (95%CI, 2.68-6.53) and 6.83 (95%CI, 4.14-1.29) in the unchanged and deteriorated groups, respectively. In girls, the ORs of elevated BP, stage 1 HTN, and stage 2 HTN were 0.52 (95%CI, 0.45-0.58), 0.40 (95%CI, 0.33-0.49), and 0.31 (95%CI, 0.20-0.46), respectively, showing sex differences (p < 0.001).

**Table 4:** Odds ratio and 95%CI for relationships between high BP and changes in the BMI category and sex aged 15 years (end of the follow-up) CI: confidence interval; BP: blood pressure; BMI: body mass index; stage 1 HTN: stage 1 hypertension; stage 2 HTN: stage 2 hypertension; OR: odds ratio. †Classified according to the Clinical Practice Guidelines for Screening and Management of High Blood Pressure in Children and Adolescents (2017) [7]. ‡Maintained group: those who maintained a normal BMI between aged 12 and 15 years; improved group: those in whom the category improved from extremely obese, obese, or overweight aged 12 years to a normal BMI aged 15 years and those in whom the category improved to obese or overweight; unchanged group: those in whom the BMI category remained the same between aged 12 and 15 years; deteriorated group: those in whom the category was a normal BMI, overweight, or obese aged 12 years but deteriorated aged 15 years. §Adjusted for sex, survey years, and baseline systolic blood pressure (as continuous); however, when sex was used as the independent variable, it was adjusted for survey years, baseline systolic blood pressure, and follow-up BMI (as continuous).

Variables	N	Elevated BP^†^ (19%)		Stage 1 HTN^†^ (7.5%)		Stage 2 HTN^†^ (1.9%)	
		OR (95%CI)^§^	p-value	OR (95%CI)^§^	p-value	OR (95%CI)^§^	p-value
		Number of subjects (%)		Number of subjects (%)		Number of subjects (%)	
Change in the BMI category							
Maintained group^‡^	5,688	1 (reference)		1 (reference)		1 (reference)	
		996 (17.5)		319 (5.6)		50 (0.9)	
Improved group^‡^	484	1.07 (0.79-1.24)	0.092	1.64 (1.21-2.22)	0.002	-	-
		97 (20)		62 (12.8)		6 (1.2)	
Unchanged group^‡^	569	1.24 (1.01-1.53)	0.043	1.98 (1.51-2.58)	<0.001	4.19 (2.68-6.53)	<0.001
		156 (27.4)		95 (16.7)		48 (8.4)	
Deteriorated group^‡^	349	1.51 (1.17-1.94)	0.001	2.30 (1.66-3.17)	<0.001	6.83 (4.14-11.29)	<0.001
		100 (28.7)		55 (15.8)		29 (8.3)	
Sex^§^							
Boy	3,580	1 (reference)		1 (reference)		1 (reference)	
		838 (23.4)		361 (10.1)		98 (2.7)	
Girl	3,510	0.52 (0.45-0.58)	<0.001	0.40 (0.33-0.49)	<0.001	0.31 (0.20-0.46)	<0.001
		511 (14.6)		170 (4.8)		35 (1)	

## Discussion

To the best of our knowledge, this follow-up study was the first to investigate the relationship between HBP and changes in the BMI category in pubertal Japanese children. Even within a short period of three years, changes in the BMI category were identified, and their effects on BP were demonstrated. The results obtained strongly implicated the BP category at the age of 15 years in changes in the BMI category. Since HBP in childhood has been associated with CVD TOD in young adults [22], similar conditions may be applied to present and future morbidities. The present results were consistent with the findings reported by Parker et al., showing a strong positive correlation between changes in BMI and BP percentiles, even in a short period (median: 3.1 years) [5].

During the three-year follow-up in the present study, 1.4% of children newly developed stage 2 HTN (Table 3). In a previous large-scale study, 0.3% of children developed HTN [5]. This discrepancy may be explained by an overestimation in this study because primary health checkup data were used, and HBP was diagnosed according to a single BP measurement. The observed increase in SBP was consistent with previous findings showing a rapid increase in SBP during peak growth in adolescence [23]. Moreover, 49% of children who newly developed stage 2 HTN were classified at baseline as overweight, obese, or extremely obese. Sarganas et al. found that 50% of children who were obese at baseline were hypertensive at the end of the follow-up [24]. Shirasawa et al. found no significant changes in BP in obese grade 7 students during the period from 1997 to 2010 [11]. Based on these findings, we concluded that the trend in BP in children over the years is predictive of disease prevalence and that body composition should be considered when discussing the trend of BP in school-aged children.

According to a study conducted in China (2013) [25] using AAP 2017 [7], stage 1 and stage 2 HTN were detected in 16.7% of children aged 6-12 years and in 7.9% of those aged 13-17 years. In another study performed in the United States on children aged 10-17 years (2000-2017), the prevalence of elevated BP was 16.3%, while that of stage 1 and stage 2 HTN was 13% in the initial screening and 2.3% by the third screening, and approximately 4% of children were lost to follow-up [26]. The values obtained in the initial screening were similar to those in the present study.

When JSH 2019 evaluation criteria [10] were applied, HTN was detected in 0.9% (95%CI, 0-10.58) of boys and 2% (95%CI, 0.1-9.1) of girls aged 12 years and in 5.5% (95%CI, 2.7-9.7) of boys and 1.2% (95%CI, 0-10.62) of girls aged 15 years. Song et al. reviewed the prevalence of HTN and demonstrated that in 2000, 3.47% (95％CI, 2.36-5.08) of children aged 12 years and 4.45% (95％CI, 3.06-6.44) of children aged 15 years had HTN [4]. HTN prevalence in our study cohort was relatively lower compared to the study, noting that the study did not include Japanese children.

In the present study, BP levels and the prevalence of HBP at the end of the follow-up were higher in boys (Tables 1, 3, 4), which is consistent with the findings reported by Hardy et al. (8-17 years) [27] and Yang et al. (6-17 years) [28], indicating a sex difference.

Regarding the sex difference in BP during this period, Jiang et al. investigated the relationship between the distribution of insulin-like growth factor-1 (IGF-1) and BP in boys and girls aged 11-18 years and showed that IGF-1 was associated with both SBP and DBP in boys, but not in girls, which may partially explain the different relationship with BP in both sexes [29]. Among boys in this period, body weight and BP management, which have been linked to the hypotensive effects of body weight loss [30], were more important toward adulthood.

There were several limitations that need to be addressed. Primary health checkup data were used in this study, which conflicted with the recommendation that BP needs to be measured on three different occasions [3,9,10], possibly resulting in the overestimation of BP increases in this population. Furthermore, the participants in this study were children living in only one region in Japan. Analyses using the LMS method revealed that height and body weight z-scores were small and physically representative of children aged 12 and 15 years in Japan (Table 1). However, the validity of generalizing the results remains unclear.

Another limitation is that we evaluated BMI and BP by classifying them based on the CDC criteria for children and adolescents in the United States and criteria for HBP screening by sex, age, and height. The present results revealed that the median BMI, SBP, and DBP z-scores were not high and may be applied to Japanese children as evaluation criteria.

Considering the differences in body type, race, lifestyle, eating habits, and environment, specific criteria and classification methods should be determined for Japanese children based on scientific evidence derived from large-scale epidemiological studies on the population. In addition, factors that may have affected BP other than BMI, including eating habits, lifestyle, family environment, family history, and socioeconomic status, were not assessed in the present study, and the effects of these factors on BP cannot be ignored. Therefore, these issues require careful evaluations to generalize the study results.

## Conclusions

While obesity is known to affect BP, the results obtained on the relationship between HBP and BMI category changes during puberty suggest the importance of controlling BP increases early in life before the onset of obesity. BP measurements in annual health checkups need to be continued at Japanese schools. The establishment of evidence-based criteria and classification methods for BP and BMI in Japanese children will play a role in promoting health and preventing illness in children.

## Appendices

Table 5 shows the baseline values and HBP percentages in the primary health checkup each year during the survey and reference values by the Japanese Society of Hypertension (JSH) (2019).

**Table 5:** Ninety-fifth percentile values for SBP/DBP in annual health checkups for the prevention of lifestyle-related diseases among children between 1993 and 2000 and reference values for hypertension in Japanese children in the Japanese Society of Hypertension guidelines 2019 SBP: systolic blood pressure; DBP: diastolic blood pressure; BP: blood pressure; CI: confidence interval. †The Japanese Society of Hypertension guidelines for the management of hypertension (JSH) (2019) [10]. ‡Numbers of subjects in health checkups. Boys: at baseline (total: 5,342), 1993: 1,103; 1994: 1,081; 1995: 1,079; 1996: 1,037; 1997: 1,042; at the end of the follow-up (total: 4,077), 1996: 874; 1997: 776; 1998: 817; 1999: 816; 2000: 794. Girls: at baseline (total: 5,006), 1993: 1,056; 1994: 974; 1995: 1,003; 1996: 978; 1997: 995; at the end of the follow-up (total: 3,822), 1996: 821; 1997: 755; 1998: 769; 1999: 724; 2000: 753.

Follow-up period	Number of participants	Baseline		Reference values for hypertension in children in the 2019 guidelines^†^	End of the follow-up		Reference values for hypertension in children in the 2019 guidelines^†^
		SBP/DBP (mmHg)	High BP % (95%CI)		SBP/DBP (mmHg)	High BP % (95%CI)	
Boy^‡^							
1993-1996	807	134/71	7.7 (2.4-17.5)	SBP ≥ 140 mmHg and/or DBP ≥ 85 mmHg	137/73	9.4 (3.9-18.4)	SBP ≥ 140 mmHg and/or DBP ≥ 85 mmHg
1994-1997	588	135/72	8 (2-20.1)		139/76	7.5 (1.6-19.9)	
1995-1998	748	132/71	8.8 (3.1-18.6)		137/74	8.8 (3.1-18.6)	
1996-1999	726	133/70	8.5 (3.5-19.2)		137/76	8.7 (2.9-18.8)	
1997-2000	711	132/70	9.4 (3.5-19.2)		143/82	9.3 (3.5-19.1)	
Overall	3,580	-	8.5 (5.6-12.2)		-	8.8 (5.9-12.5)	
Girl^‡^							
1993-1996	746	134/72	8.3 (2.8-18.2)	SBP ≥ 135 mmHg and/or DBP ≥ 80 mmHg	133/72	8.8 (3.1-18.6)	SBP ≥ 140 mmHg and/or DBP ≥ 85 mmHg
1994-1997	674	133/72	9.3 (3.3-19.5)		132/71	6.7 (1.4-18.3)	
1995-1998	690	133/73	8.1 (2.5-18.6)		134/74	7.8 (2.2-18.6)	
1996-1999	689	132/71	7.3 (1.8-18.5)		132/73	8 (2.2-18.9)	
1997-2000	711	133/73	8 (2.5-18.4)		135/80	9.6 (3.8-19.2)	
Overall	3,510	-	8.2 (5.3-12)		-	8.2 (5.3-12)	
